# Fourth Generation of Next‐Generation Sequencing Technologies: Promise and Consequences

**DOI:** 10.1002/humu.23051

**Published:** 2016-08-10

**Authors:** Rongqin Ke, Marco Mignardi, Thomas Hauling, Mats Nilsson

**Affiliations:** ^1^ School of Biomedical SciencesHuaqiao UniversityQuanzhouFujian362021China; ^2^ Department of Information TechnologyCentre for Image AnalysisScience for Life LaboratoryUppsala UniversityUppsalaSE‐75105Sweden; ^3^ Department of BioengineeringStanford UniversityStanfordCalifornia75105; ^4^ Department of Biochemistry and BiophysicsScience for Life LaboratoryStockholm UniversitySolnaSE‐171 21Sweden

**Keywords:** next‐generation sequencing, in situ sequencing, single cell sequencing, spatial gene expression, multiplex in situ RNA detection

## Abstract

In this review, we discuss the emergence of the fourth‐generation sequencing technologies that preserve the spatial coordinates of RNA and DNA sequences with up to subcellular resolution, thus enabling back mapping of sequencing reads to the original histological context. This information is used, for example, in two current large‐scale projects that aim to unravel the function of the brain. Also in cancer research, fourth‐generation sequencing has the potential to revolutionize the field. Cancer Research UK has named “Mapping the molecular and cellular tumor microenvironment in order to define new targets for therapy and prognosis” one of the grand challenges in tumor biology. We discuss the advantages of sequencing nucleic acids directly in fixed cells over traditional next‐generation sequencing (NGS) methods, the limitations and challenges that these new methods have to face to become broadly applicable, and the impact that the information generated by the combination of in situ sequencing and NGS methods will have in research and diagnostics.

## Introduction

Tissue is constituted of a complex organization of different cell types that are tightly regulated by the interplay of individual cells within it. Thus, to better understand the physiological and pathological status of normal or diseased tissue, decomposition of the complexity by single cell analysis is necessary. In recent years, next‐generation sequencing (NGS)‐based single cell RNA sequencing (scRNA‐seq) technology has been proven to be a powerful tool for different applications, for example, classifying cell subpopulations [Usoskin et al., 2015], identifying rare cells [Grün et al., 2015], and defining cell lineage [Blakeley et al., 2015], providing new biological insights into the composition of tissues, the dynamics of transcription and the regulatory network of different genes [Deng et al., 2014; Shalek et al., 2014; Brennecke et al., 2015; Hanchate et al., 2015].

Most of the scRNA‐seq methods rely on separation of single cells from tissue by enzymatic or mechanical dissociation resulting in loss of spatial information. Laser‐assisted microdissection is a method to capture cells of interest through direct visualization under the microscope [Emmert‐Buck et al., 1996]. Single cells are then subjected to downstream analysis, providing analyzed results that can then be linked to the spatial localization in the original tissue [Hölscher and Schneider, 2008]. However, the contextual information is limited because only the target cells are analyzed but not their surrounding neighbor cells that form the microenvironmental niche of the target cells [Miller et al., 2014]. Alternatively, whole tissue can be subdivided into smaller sections, followed by analysis of all sections [Hawrylycz et al., 2012]. Another approach to link spatial information to bulk sequencing data employs sequencing of serial consecutive sections in different directions to enable computational reconstruction of spatial expression patterns [Junker et al., 2014; Wu et al., 2016]. However, these approaches require multiple identical samples to provide spatial resolution in all dimensions. Another way is to use computational methods that enable mapping the information generated by scRNA‐seq data to the tissue of origin by using previous gene expression data obtained from in situ hybridization (ISH) as reference [Achim et al., 2015; Satija et al., 2015]. With these approaches, authors were able to position cells with scRNA‐seq data to their locations within the tissue. The major limitation of these methods is the dependence on a priori knowledge about spatial gene expression patterns of the specimen. Therefore, inherent to the method, it is limited to tissues with organized and reproducible texture, potentially excluding most tumors or any specimen, which is highly heterogeneous and unique in nature.

The recent developments of the fourth generation of sequencing methods, such as in situ sequencing (ISS), hold great promises as they enable highly spatially resolved transcriptomics regardless of the specimen by sequencing nucleic acids directly in cells and tissue [Ke et al., 2013; Lee et al., 2014]. ISS methods rely on previously described NGS sequencing chemistries, and as such allow for robust detection even of single‐nucleotide variations. ISS is complemented by other spatially resolved multiplex transcriptomics technologies that are based on classic ISH protocols, combined with combinatorial or sequential labeling schemes or combinations thereof. An interesting alternative to ISS or ISH, termed spatial transcriptomics, employs a combination of in situ transcript mapping and ex situ transcript identification by NGS. The following paragraphs will discuss functional principles; key strengths and weaknesses of ISS‐based and other spatially resolved transcriptomics technologies.

### Massively Parallel Spatially Resolved Sequencing

Joakim Lundeberg and colleagues developed a novel method, which is now offering early access through their startup company, Spatial Transcriptomics [Ståhl et al. 2016]. In this new technology, a fresh‐frozen tissue section is deposited onto a chip containing an array of 100 μm features of unique sequence‐barcoded oligo‐dT capture probes equipped with sequencing adaptors. After imaging the tissue to record the positions of the cells relatively to the array, the sample is permeabilized and the mRNA diffuse onto the array of capture probes. The probes are then used as primers for cDNA synthesis ”on‐chip,” generating a sequencing library that can subsequently be retrieved and analyzed by NGS. Each read can then be mapped back to a feature based on its spatial barcode. While not offering single cell resolution in its current state, spatially defined regions can be analyzed transcriptome wide at a high throughput.

### Single Cell In Situ Transcriptomics

Several sequential and combinatorial labeling and imaging approaches based on single molecule fluorescence ISH (smFISH) have been developed in order to expand the throughput of in situ RNA detection. Sequential staining methods can rely on one or multiple fluorophores. In the approach developed by Lubeck et al. (2014), sets of 24 detection probes labeled with the same dye for a given transcript are hybridized, imaged, and stripped with DNaseI. In subsequent cycles, the same sets of probes are labeled with different dyes according to a combinatorial scheme that will create a unique sequence of labels between hybridization cycles. Although >95% of all mRNA molecules in a cell can be detected due to the high efficiency of the hybridization reaction the colocalization rate, which is a prerequisite for accurate and effective multiplexing, seemed to be relatively low (77.9% ± 5.6% between the first two hybridizations).

Chen et al. (2015b) developed a technology called multiplexed error‐robust fluorescence in situ hybridization (MERFISH) that is capable of determining the identity, the copy numbers, and locations of thousands of RNA molecules inside a single cell. MERFISH relies on binary labeling, that is, target RNAs are either fluorescence positive or negative for any given imaging cycle. In their method, encoding probes that contain target‐specific hybridization sequences extended with readout sequences are first hybridized to target RNAs. In each imaging cycle, a subset of fluorophore‐conjugated readout probes is hybridized to a subset of encoding probes. RNAs that fluoresce in this cycle are assigned with a “1,” whereas others are assigned with a “0.” Between imaging cycles, the fluorescence from the previous cycle are photobleached. After 14 or 16 rounds of hybridization, unique combinations of readout probes generate a 14‐bit or 16‐bit code that identifies different genes. However, as the hybridization rounds increase, the calling rate decreases and the error rate increases. In order to address this issue, the authors introduced the Hamming distance, which is used in telecommunication, to enable detection and correction of encoding errors in RNA barcodes, resulting in an error‐robust barcoding scheme. Up to 1,001 distinct mRNA species were identified using this approach. Furthermore, by analyzing fluctuations in the expression levels of different genes, gene regulatory networks were mapped and novel functions for many unannotated genes could be predicted.

The above‐described smFISH methods require high‐resolution optical imaging setups and are consequently limited in throughput. Therefore, they have only been demonstrated in single cells and not in whole tissue sections.

### In Situ RNA Sequencing

Two pioneering methods have been developed that utilize second‐generation NGS chemistry to sequence single RNA molecules directly in fixed cells and tissues [Ke et al., 2013; Lee et al., 2014].

ISS is potentially adaptable to diagnostics applications, which typically rely on tissue material that has been preserved by extensive cross‐linking followed by paraffin embedding: the formalin‐fixed, paraffin‐embedded (FFPE) tissues. Cross‐linking leads to covalent modification of nucleic acid bases as well as strand cleavage [Evers et al., 2011], thereby shortening the length of intact RNA molecules that can be extracted from such specimens. Although cross‐links are to some extent reversible, it can still be difficult to obtain annotatable reads from such specimens. In addition, scRNA‐seq cannot be performed since cross‐linking precludes the isolation of individual cells from FFPE tissue. ISS interrogates short ≤40 nucleotide long motifs and does not require isolation of nucleic acids from the tissue material.

**Figure 1:**
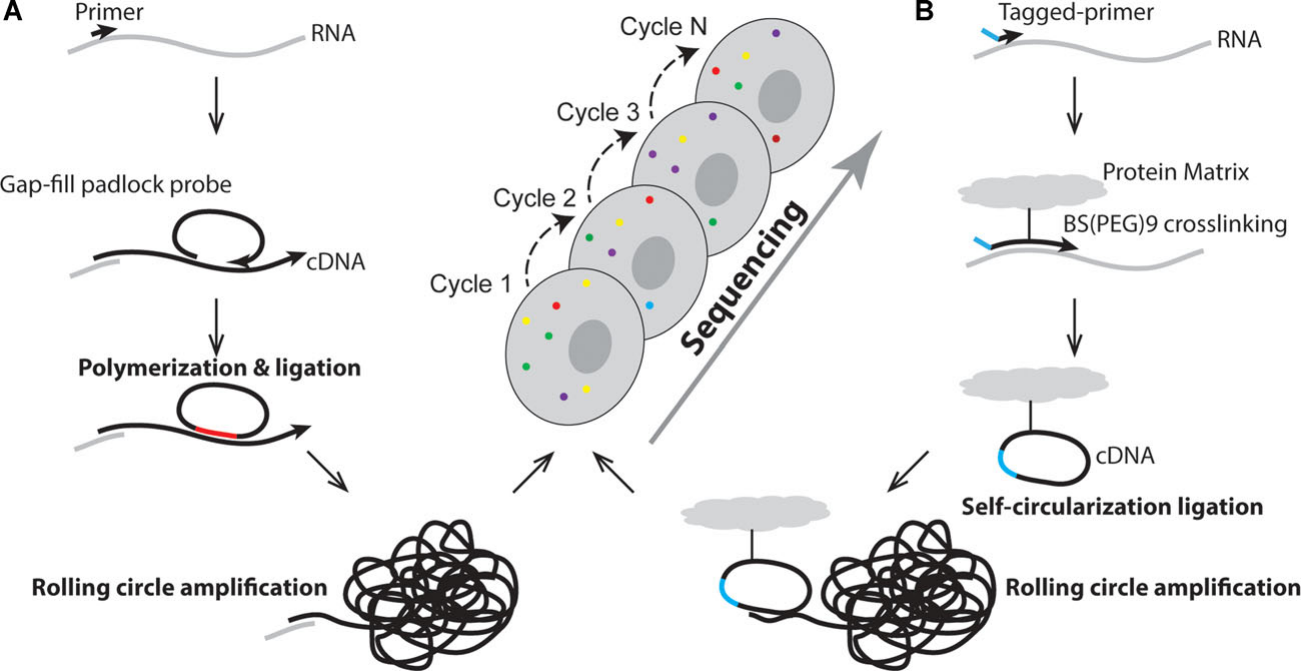
Schematic illustration of in situ sequencing. **A**: Padlock probe‐based in situ sequencing. RNA is first converted to cDNA in situ within cells or tissue using LNA‐modified primers or random primers, followed by removal of RNA strand and hybridization of a modified padlock probe, leaving a gap between the two ends of the probe. The gap, which is the target of interest for sequencing, is then filled by DNA polymerization and then DNA ligation to form a complete DNA circle. Rolling circle amplification is performed to clonally amplify the DNA circle, generating rolling circle amplification product that is subjected to sequencing by ligation chemistry. **B**: FISSEQ. Complementary DNA is first generated by in situ reverse transcription using tagged random primer and dNTP mixed with aminoallyl deoxyuridine 5′‐triphosphate (dUTP). The resulting cDNA is then cross‐linked to the cellular matrix with a cross‐linking reagent to ensure immobilization of cDNA, therefore preserving the spatial information. The newly synthesized cDNA is self‐circularized to form a DNA circle using CircLigase, followed by clonal amplification using rolling circle amplification. Finally, SOLiD sequencing chemistry is performed to sequence the target fragment that was encircled.

Our group has developed methods for in situ single RNA molecule detection with single nucleotide resolution by using padlock probes combined with rolling circle amplification (RCA) [Larsson et al., 2004, 2010]. Padlock probes are linear oligonucleotide probes that become circularized by a DNA ligase upon specific hybridization to a target sequence [Nilsson et al., 1994]. RCA is a DNA circle‐specific method that clonally amplifies the sequence of the DNA circle, forming a submicron‐sized blob of DNA (or DNA nanoballs) locally preserved at the site of the DNA circle formation [Lizardi et al., 1998]. We applied this strategy to generate in situ amplified targeted sequencing libraries and subjected them to NGS chemistry. This way, fragments of single RNA molecules or molecular barcodes can be sequenced in situ within morphologically preserved cells and tissue [Ke et al., 2013] (Fig. 1A). In our method, RNA molecules are first fixed to their natural environment using paraformaldehyde, followed by in situ reverse transcription of RNA into cDNA by using target‐specific lock nucleic acid‐modified primers, or nontarget‐specific random primers. Thereafter, padlock probes are designed to hybridize with their ends flanking the target‐of‐interest. The gap is then filled by DNA polymerization and ligation, thus circularizing the probe. By using this approach (gap‐fill approach), a short fragment of cDNA is cloned into the DNA circle, which is then clonally amplified by RCA, generating substrates for NGS chemistry in situ. We applied the sequencing‐by‐ligation (SBL) approach developed by George Church lab and at Complete Genomics, to sequence the cloned fragment [Shendure et al., 2005; Drmanac et al., 2010]. With this approach, we were able to distinguish an SNV between human and mouse cell  *β*‐actin (ACTB) mRNA in cultured cells. We also sequenced short fragments of ACTB and HER2 transcripts in a breast cancer tissue, and sequenced codon 12 and 13 of the KRAS transcript to detect a KRAS mutation in rare (one in 1,000) KRAS mutation‐positive cancer cells spiked into a background of KRAS‐negative cells.

Sequencing a short RNA fragment of four to six bases is good for the detection of SNVs and small deletions, but is not needed for the purpose of just detecting and quantifying a large number of transcripts. Therefore, in a second approach, we used our ISS protocol to sequence a molecular barcode of four bases in the nontarget hybridization part of the padlock probes, providing a coding capacity of up to 256 (4^4^) different transcripts. We demonstrated this multiplexed mRNA detection approach targeting 39 different genes in frozen tissue sections of one ER‐negative and two ER‐positive breast tumors. The set of genes include the Oncotype DX 21 gene expression panel used to predict distant tumor recurrence in breast cancer patients [Sparano and Paik, 2008]. We were able to obtain in situ expression patterns of 31 of the genes, and we observed clearly distinct expression patterns between the HER2‐positive cancer cells and VIM‐positive stromal cells. When compared with published RNA sequencing data from a range of tissues and cell lines, the cancer cell profile had showed best match with a breast cancer‐derived cell line, whereas the stromal profile matched normal breast tissue RNA sequencing data best. In another study, our ISS method was used to determine the distribution of TMPRSS2‐ERG fusion transcripts, together with somatic point mutations and gene expression levels of some biomarkers in prostate tumors [Kiflemariam et al., 2014].

Lee et al. (2014) developed a similar in situ expression profiling method termed fluorescent ISS (FISSEQ), which generates random libraries, in contrast to barcoded probes for gene panels used in our targeted ISS (Fig. 1B). Random hexamers with a sequencing primer tag are used to initiate in situ reverse transcription, converting RNA into cDNA. The resultant cDNA is then circularized to form a DNA circle using CircLigase that does not need a ligation template. During reverse transcription, aminoallyl deoxyuridine 5′‐triphosphate (dUTP) is incorporated into the cDNA and subsequently cross‐linked to the cell protein matrix by using a cross‐linking reagent called BS(PEG)9, preventing the cDNAs from diffusing away. After RCA, the RCA products were sequenced by oligonucleotide ligation and detection (SOLiD) chemistry. Lee et al. (2014) showed that the FISSEQ library preparation step not only works in cultured cells, but also in tissues such as mouse embryo and adult brain sections and whole‐mount Drosophila embryos. However, sequencing was not performed in these tissues. Lee et al. (2014) examined RNA expression and localization in human primary fibroblasts with a simulated wound‐healing assay. By applying FISSEQ with 30‐base read length, they obtained 156,762 reads covering 8,102 annotated genes. However, while theoretically covering the whole transcriptome, FISSEQ in practice appears to be limited in sequencing depth, since the vast majority (>80%) of amplicons represent ribosomal RNA [Lee et al., 2015]. The sensitivity of targeted ISS has been estimated to be two orders of magnitude higher than FISSEQ for a given gene [Lee et al., 2015]. A possible reason for this is that target‐specific library construction enables exclusion of highly expressed transcripts, thereby avoiding signal overcrowding. To overcome the optical limitation and signal overcrowding problem, Lee et al. (2015) developed a partition sequencing approach by using sequencing primers that have one or several bases extended into the sequencing target, reducing the number of substrates that can be sequenced per reaction, but on the other hand, multiple sequencing reactions using different anchor primers have then to be performed to sequence all substrates.

### Challenges and Limitations of ISS

Although ISS technology represents a promising tool, many technical aspects need to be addressed before it can be broadly applied. The main bottlenecks are sample imaging, the relatively low efficiency of molecular processes, data handling, and interpretation. Both of the two current ISS methods exploit optical fluorescence imaging‐based sequencing chemistry as readout strategy. Some biological samples exhibit high levels of autofluorescence that may affect the fluorescent signal during image analysis. To remove autofluorescence, methods such as light irradiation, used in conventional FISH, can be applied to ISS [Neumann and Gabel, 2002], or one of the many tissue clearing protocols recently developed [Chung and Deisseroth, 2013; Yang et al., 2014].

An alternative approach is to use dyes that do not spectrally overlaps with the autofluorescence, or to use time‐resolved fluorescence dyes [Suhling et al., 2005]. Another limitation is the dimension of the sequencing substrates in relation to the size of cells. A cell is limited in both the projected 2D area and its 3D volume, setting the limit for the number of optical sequencing reactions that can take place within a cell. This number is depending on the size of the sequencing substrate (the RCA products), and the optical resolution of the imaging system. For padlock‐based ISS, the maximum number of RCA products per cell in a breast tumor section was estimated to about 100. This number could be increased about four times by “partition sequencing” used in the FISSEQ approach. New approaches, such as expansion microscopy [Chen et al., 2015a], may overcome this physical limitation.

Data handling and interpretation of ISS also faces challenges. With ISS, sequence information as well as its spatial information can either be recorded in 2D or 3D. The imaging process generates large amount of data, about 10 GB per sequencing cycle to cover an area of 50 mm^2^ at 20x magnification (with the system used in Ke et al., 2013). This is in the same order of magnitude of NGS technologies that employ imaging read out (i.e., Illumina platforms), and will thus require a way to store data similar to the data compression developed for NGS [Daily et al., 2010; Fritz et al., 2011; Deorowicz and Grabowski, 2013].

### Promise and Consequences

Fourth‐generation sequencing technologies, especially the ISS, combine traditional imaging analysis techniques and the state‐of‐the‐art NGS technologies to offer new opportunities for studying tissue heterogeneity. For example, several recent projects that aim to unravel the function of brain rely on spatially resolved transcriptomics to map the complexity of this organ (Wellcome Trust funded Neuromics project, IARPA funded Brain Mapping Consortium). According to recent scRNA‐seq studies, the brain consists of hundreds of subtypes of the different cell types. Understanding how they are connected and where they are located will, without doubt, enhance our understanding of neurological disorders and how the mind works in general. Cancer Research UK has named “Mapping the molecular and cellular tumor microenvironment in order to define new targets for therapy and prognosis” one of the grand challenges in tumor biology. Our laboratory engages in several projects that ultimately aim at relating treatment response to spatial transcription profiles of tumors.

**Figure 2:**
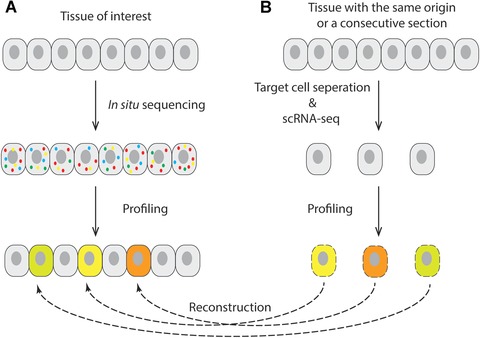
Spatial reconstruction of single cell gene expression data using in situ sequencing and scRNA‐seq. **A**: In situ sequencing is performed on tissue of interest to generate gene expression data that are used to profile different cell types. **B**: Single cell obtained from a tissue with the same origin (same morphology) or from a consecutive section are subjected to scRNA‐seq. After profiling the scRNA‐seq data, cells that match the in situ sequencing gene expression patterns are placed to their corresponding positions to reconstruct the spatial organization of the tissue.

Although current ISS fails to provide whole transcriptome profiles, mainly due to its limitation in sensitivity and molecular crowding, it has a great potential to become a tool that synergistically can be combined with scRNA‐seq technology. For instance, scRNA‐seq can identify and define cell‐types among a population of cells isolated from a tissue. The expression profile from these molecularly defined cell types can then be compared and matched to expression profiling generated by ISS on the same type of tissue or, even better, on a consecutive section of the same tissue. In this way, the cell types and states defined by scRNA‐seq can be mapped to their positions in the tissue, providing a more complete picture of spatial gene expression, whereas the ISS provides positional information for millions of cells based on a selection of biomarkers identified by scRNA‐seq, whereas the scRNA‐seq provides deep information about the transcriptional state of the molecularly defined cell types (Fig. 2).

In conclusion, fourth‐generation ISS provides a different and complementary paradigm to the analysis of genome and transcriptome using other NGS technologies. It may be possible to become a standard method for the sequencing of tissue samples if technical obstacles are overcome through further development of the technologies [Crosetto et al., 2015]. Furthermore, new generation biomarkers that contain spatial information may be discovered and come to a point where large‐scale ISS of tissue samples will be deployed for diagnostics.
